# Lipids and Composition of Fatty Acids of *Saccharina latissima* Cultivated Year-Round in Integrated Multi-Trophic Aquaculture

**DOI:** 10.3390/md13074357

**Published:** 2015-07-15

**Authors:** Gonçalo S. Marinho, Susan L. Holdt, Charlotte Jacobsen, Irini Angelidaki

**Affiliations:** 1 Department of Environmental Engineering, Technical University of Denmark, Miljøvej, Building 113, Kgs. Lyngby 2800, Denmark; E-Mail: gosm@env.dtu.dk; 2 National Food Institute, Technical University of Denmark, Søltofts Plads 221, Kgs. Lyngby 2800, Denmark; E-Mails: suho@food.dtu.dk (S.L.H.); chja@food.dtu.dk (C.J.)

**Keywords:** seaweed, IMTA, epiphytes, *n*-3, FAME, PUFA, EPA, DHA

## Abstract

This study is evaluating the seasonal lipid and fatty acid composition of the brown seaweed *Saccharina latissima*. Biomass was sampled throughout the year (bi-monthly) at the commercial cultivation site near a fish farm in an integrated multi-trophic aquaculture (IMTA) and at a reference site in Denmark (2013–2014). Generally, there was no difference in the biomass composition between sites; however, significant seasonal changes were found. The lipid concentration varied from 0.62%–0.88% dry weight (DW) in July to 3.33%–3.35% DW in November (*p* < 0.05) in both sites. The fatty acid composition in January was significantly different from all the other sampling months. The dissimilarities were mainly explained by changes in the relative abundance of 20:5*n*-3 (13.12%–33.35%), 14:0 (11.07%–29.37%) and 18:1*n*-9 (10.15%–16.94%). Polyunsaturated fatty acids (PUFA’s) made up more than half of the fatty acids with a maximum in July (52.3%–54.0% fatty acid methyl esters; FAME). This including the most appreciated health beneficial PUFA’s, eicosapentaenoic (EPA; 20:5*n*-3) and docosahexaenoic acid (DHA; 22:6*n*-3), but also arachidonic (ARA) and stearidonic acid (SDA), which are not found in land vegetables such as cabbage and lettuce. Compared to fat (salmon) and lean fish (cod) this seaweed species contains higher proportions of ARA and SDA, but lower EPA (only cod) and DHA. Conclusively, the season of harvest is important for the choice of lipid quantity and quality, but the marine vegetables provide better sources of EPA, DHA and long-chain (LC)-PUFA’s in general compared to traditional vegetables.

## 1. Introduction

Humans and other vertebrates lack the ability to synthesize linoleic (18:2*n*-6) and α-linolenic acid (18:3*n*-3) [1]. Moreover, conversion of the precursor α-linolenic acid to the physiologically essential eicosapentaenoic acid (EPA; 20:5*n*-3) and docosahexaenoic acid (DHA; 22:6*n*-3) by humans is restricted [2]. Similarly, although fish have the ability to elongate and desaturate α-linolenic acid into long-chain polyunsaturated fatty acids (LC-PUFA), that capacity is generally limited, particularly in marine species [3,4]. Thus, LC-PUFA such as EPA and DHA must be supplied in the diet. Currently, oily fish and fish oils remain the main sources of *n*-3 and *n*-6 LC-PUFA [5].

Appropriate intake of *n*-3 LC-PUFA reduces the risk of cardiovascular diseases, cancer, depression and mental illness and promotes the development of the nervous system [6,7]. In addition, a balanced dietary *n*-6/*n*-3 ratio has been recommended by World Health Organization (WHO) to prevent inflammatory, cardiovascular and nervous system disorders [1]. Moreover, the *n*-3 LC-PUFA are essential dietary nutrients for good growth and survival of fish [8,9].

The established health benefits of *n*-3 LC-PUFA, together with the predictable depletion of fish stocks, the main source of such fatty acids, have driven the search for non-conventional biomass sources as alternative sources of these fatty acids [1,10]. In this context, both micro and macroalgae (seaweeds) have been pointed out as potential candidates for the provision of LC-PUFA. Although the lipid content of seaweed is relatively low, they contain higher levels of PUFA than land vegetables [11]. Seaweeds constitute a huge and renewable resource with a lipid profile rich in LC-PUFA, which has attracted high interest [12,13,14]. Arachidonic acid (ARA; 20:4*n*-6) and EPA are the most abundant PUFA in red and brown seaweeds. On the other hand, green seaweeds as well as terrestrial plants contain lower levels of these PUFA [1,10,12].

The most important seaweeds used as human food include the red seaweed *Porphyra* spp. (nori), and the brown seaweeds *Laminaria japonica* (kombu) and *Undaria pinnatifida* (wakame) with a combined aquaculture production of 9.6 million tonnes in Asia in 2012 [15]. Similarly, *Saccharina latissima* has the potential to be produced sustainably through commercial aquaculture [16,17,18]. The estimated yields of *S. latissima* cultivated in Scotland, Spain and Denmark, as monoculture or in integrated multi-trophic aquaculture (IMTA), ranged from 5.1 to 340 t fresh weight ha^−1^ over a growing season [18,19,20]. In IMTA systems, organisms from different trophic levels are co-produced, and waste nutrients from fed species (e.g., fish and shrimp) are assimilated and converted into valuable biomass by the extractive species (e.g., seaweed), providing environmental and potentially economic benefits (see reviews by Neori *et al.*, and Chopin *et al.* [21,22]). Seaweeds cultivated in IMTA systems have been reported to increase their nitrogen and protein content compared to seaweeds cultivated in reference locations in response to the nutrient loading from fish farms [23,24,25]. Environmental conditions have been shown in many studies to change the lipid concentration and fatty acid profiles of especially microalgae [26], and, furthermore, a study on a seaweed species of *Ulva* showed that limitation and, surprisingly, high levels of nitrogen also increased the lipid content and changed the profile of the major PUFA’s [27]. A hypothesis of this study is that the lipid content and fatty acid composition of *Saccharina latissima* may be different due to the possible different nutrient regimes at the two cultivation sites.

Chemical composition of seaweed, including fatty acid content and composition varies according to season, geographic area and environmental factors [10,13,28]. For instance, seaweed from cold water present generally higher PUFA content then those from warmer waters [13]. The fatty acid composition of *S. latissima* from the French Brittany coast was previously reported [12]; however, seasonal changes were not evaluated. Schmid *et al.* [10] have found marked changes in the fatty acid composition of *S. latissima* collected from natural populations at two different seasons in western Ireland. The authors emphasized the need for a better understanding of seasonal dynamics of fatty acid profile and content. The presence of epiphytes, organisms that live on the thallus of some seaweeds, which for *S. latissima* are mostly found during summer time [18], is another factor that needs to be accounted for when evaluating the potential utilization of *S. latissima* for food or feed.

Therefore, this study aimed to evaluate changes in the lipid content and fatty acid composition of *S. latissima* in response to possible differences in the nutrient regime between IMTA and reference site, and seasonal fluctuations in the environmental conditions. Changes in the lipid content and fatty acid composition of *S. latissima* (also including epiphytes when present) were determined every second month, throughout a 12-month period, and its potential use for food and feed upon different harvest times was discussed. Particular focus was placed on fatty acids with reported health benefits such as EPA and DHA.

## 2. Results

**Figure 1:**
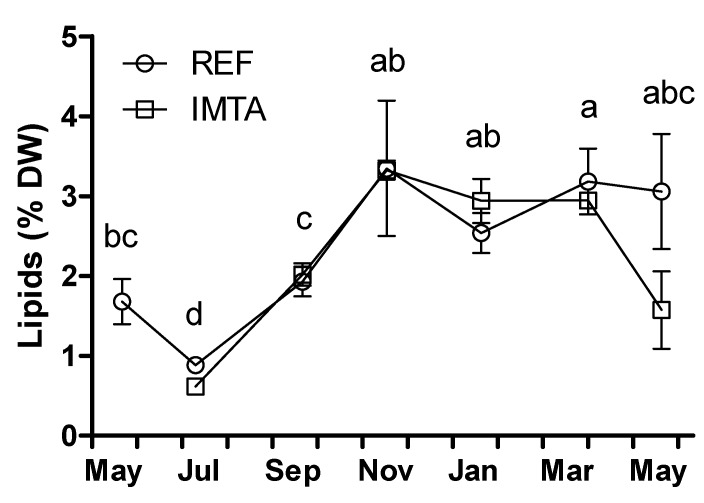
Year-round variation of lipid content (% dry weight; DW) of *Saccharina latissima* cultivated at a reference site (REF) and at an integrated multi-trophic aquaculture (IMTA) in 2013–2014. Biomass cleaned of epiphytes when present (July–November). The standard errors are presented as bars (*n* = 3), and different letters indicate significant differences between sampling months (*p* < 0.05).

**Figure 2:**
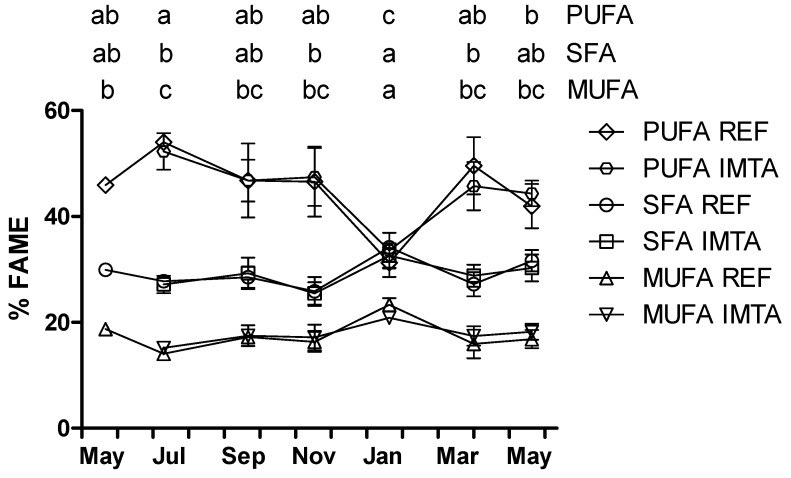
Year-round variation of polyunsaturated fatty acids (PUFA), saturated fatty acid (SFA) and monounsaturated fatty acid (MUFA) (% fatty acid methyl esters; FAME) of *Saccharina latissima* cultivated at a reference site (REF) and at an integrated multi-trophic aquaculture (IMTA) in 2013–2014. Biomass cleaned of epiphytes when present (July–November). The standard errors are presented as bars (*n* = 3), and different letters in the same row indicate significant differences between sampling months (*p* < 0.05).

Significant seasonal differences in the lipid content and fatty acid composition were found in this study (*p* < 0.05). On the contrary, the differences between sites were not of significance (*p* *>* 0.05). The lipid content varied significantly from 0.62%–0.88% dry weight (DW) in July to 3.33–3.35% DW in November (*p* < 0.05; Figure 1). Identified fatty acids ranged from 87.0% to 95.9% fatty acid methyl esters (FAME) for all the analyzed samples (Table 1). Saturated fatty acids (SFA) accounted for a minimum of 25.5%–25.9% FAME in November, while monounsaturated fatty acids (MUFA) contributed to a minimum of 14.1%–15.2% FAME in July. In January, SFA increased significantly to 32.6%–34.2% and MUFA increased to 20.9%–23.3% FAME (*p* < 0.05; Figure 2). Contrarily, polyunsaturated fatty acids (PUFA) made up to a minimum of 31.3%–33.6% FAME in January and a maximum of 52.3%–54.0% FAME in July.

**Figure 3:**
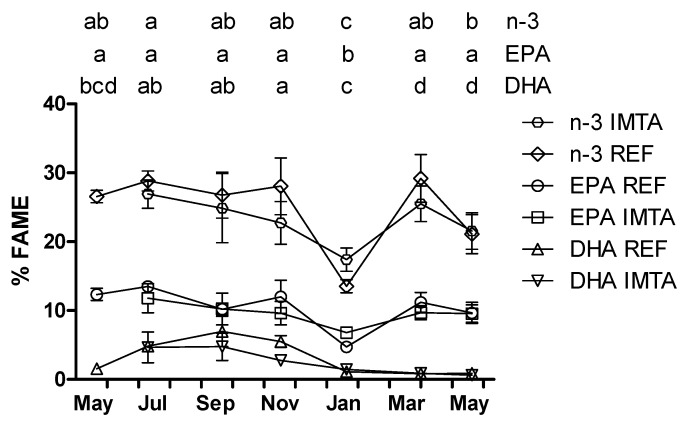
Year-round variation of *n*-3, eicosapentaenoic acid (EPA) and docosahexaenoic acid (DHA) (% FAME) of *Saccharina latissima* cultivated at a reference site (REF) and at an integrated multi-trophic aquaculture (IMTA) in 2013–2014. Biomass cleaned of epiphytes when present (July–November). The standard errors are presented as bars (*n* = 3), and different letters in the same row indicate significant differences between sampling months (*p* < 0.05).

**Figure 4:**
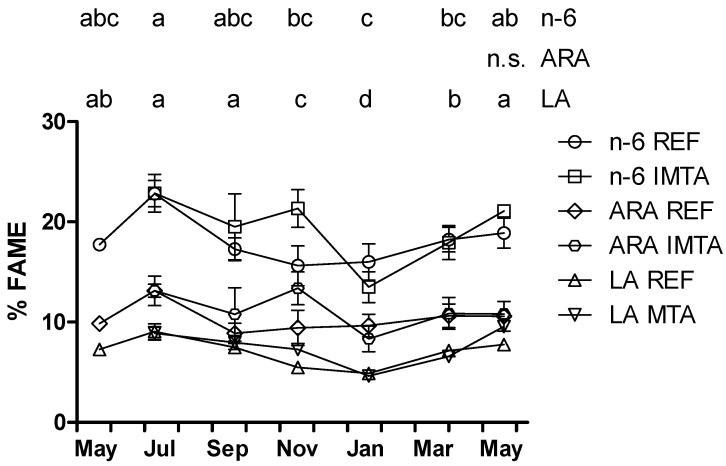
Year-round variation of *n*-6, arachidonic acid (ARA) and linoleic acid (LA) (% FAME) of *Saccharina latissima* cultivated at a reference site (REF) and at an integrated multi-trophic aquaculture (IMTA) in 2013–2014. Biomass cleaned of epiphytes when present (July–November). The standard errors are presented as bars (*n* = 3), and different letters in the same row indicate significant differences between sampling months (*p* < 0.05). n.s., not significant.

SFA palmitic acid (16:0) dominated the fatty acid composition for all sampling months representing 14.8%–19.4 % FAME (Table 1). Oleic acid (18:1*n*-9) was the most abundant MUFA ranging seasonally from 8.3% to 13.9% FAME. Of the PUFA, the *n*-3 and the *n*-6 series reached a maximum of 26.9%–28.8% and 22.8%–22.9% FAME, respectively, in July (Figure 3 and Figure 4). The *n*-3 series constituted a minimum of 13.5%–17.4% FAME in January while the *n*-6 series represented a minimum of 13.5%–15.6% FAME in November–January (*p* < 0.05). The *n*-6/*n*-3 ratio varied between 0.56 and 1.18 for all the sampling months. Eicosanoid precursor EPA was the most abundant *n*-3 with the highest values found in July (11.8%–13.5%) and the lowest values found in January (4.7%–6.8%; *p* < 0.05). The DHA content varied from a minimum of 0.6%–1.5% (January–May) to a maximum of 4.7%–7.0% in September. EPA and DHA precursor stearidonic acid (SDA; 18-4*n*-3) content varied from 4.0%–4.9% in January to 9.3%–10.9% in March. Essential fatty acid α-linolenic acid ranged from 2.6%–3.1% in November–January to 4.7%–5.4% in March. Eicosanoid precursor ARA was the most abundant *n*-6 varying seasonally from 8.3% to 13.4% FAME, but differences between months were not significant (*p* > 0.05; Figure 4). Essential fatty acid linoleic acid varied from a minimum of 4.6%–4.9% in January to 7.3%–9.6% FAME in May–September (*p* < 0.05).

**Table 1:** Year-round variation in the fatty acid composition (% fatty acid methyl esters; FAME) of *S. latissima* cultivated at both reference (REF) and integrated multi-trophic aquaculture (IMTA) sites.

Fatty Acid	May 2013	July		September		November		January		March		May 2014		Cabbage ^1^	Lettuce ^1^	Salmon ^1^	Cod ^1^
	INITIAL	REF	IMTA	REF	IMTA	REF	IMTA	REF	IMTA	REF	IMTA	REF	IMTA				
14:0	7.71 ± 0.27	8.32 ± 1.11	6.83 ± 1.24	6.13 ± 1.57	7.19 ± 1.39	6.61 ± 0.24	7.95 ± 0.59	12.66 ± 1.13	12.44 ± 0.99	11.03 ± 0.14	12.19 ± 0.49	11.94 ± 0.87	9.87 ± 1.02			4.29	
14:1	0.18 ± 0.02	0.50 ± 0.27	0.51 ± 0.14	0.35 ± 0.01	0.31 ± 0.17	0.32 ± 0.10	0.47 ± 0.14	1.96 ± 0.62	1.93 ± 0.79	1.52 ± 0.05	1.86 ± 0.19	0.88 ± 0.26	0.95 ± 0.20				
15:0	0.49 ± 0.05	0.45 ± 0.03	0.39 ± 0.01	0.46 ± 0.05	0.53 ± 0.13	0.45 ± 0.14	0.39 ± 0.08	0.59 ± 0.12	0.51 ± 0.05	0.45 ± 0.00	0.44 ± 0.05	0.60 ± 0.10	0.74 ± 0.10				
16:0	19.41 ± 1.05	16.71 ± 0.35	17.33 ± 0.45	17.87 ± 0.95	18.08 ± 1.52	16.10 ± 1.79	14.79 ± 1.07	19.27 ± 0.96	18.62 ± 1.50	14.78 ± 1.65	15.39 ± 1.40	18.04 ± 1.37	18.28 ± 1.54	23.2	17.1	17.1	33.1
16:1 (*n*-7)	5.17 ± 0.03	3.20 ± 0.82	3.84 ± 0.75	5.05 ± 0.12	4.84 ± 0.86	4.95 ± 0.72	4.60 ± 0.22	5.60 ± 0.49	4.82 ± 0.49	2.87 ± 0.51	4.37 ± 0.42	2.41 ± 0.28	3.73 ± 0.18	2.58	1.43	5.71	
16:2 (*n*-4)	0.35 ± 0.00	0.54 ± 0.13	0.65 ± 0.02	0.65 ± 0.07	0.47 ± 0.01	0.32 ± 0.13	0.34 ± 0.01	0.97 ± 0.36	1.00 ± 0.33	0.98 ± 0.40	0.91 ± 0.35	0.27 ± 0.16	0.42 ± 0.18				
16:3 (*n*-4)	0.31 ± 0.03	0.45 ± 0.04	0.45 ± 0.17	0.59 ± 0.13	0.38 ± 0.15	0.41 ± 0.07	0.20 ± 0.04	0.30 ± 0.05	0.26 ± 0.06	0.15 ± 0.01	0.20 ± 0.02	0.16 ± 0.01	0.11 ± 0.00				
17:0	0.17 ± 0.01	0.24 ± 0.05	0.21 ± 0.04	0.48 ± 0.11	0.38 ± 0.07	0.51 ± 0.11	0.37 ± 0.06	nd	nd	nd	nd	nd	nd				
16:4 (*n*-1)	0.20 ± 0.01	0.19 ± 0.08	0.15 *	0.21 ± 0.02	0.23 ± 0.05	0.86 ± 0.17	0.52 ± 0.20	0.16 ± 0.04	0.25 ± 0.03	0.15 ± 0.03	0.14 ± 0.02	0.16 ± 0.02	0.16 ± 0.01				
18:0	1.76 ± 0.38	1.69 ± 0.24	2.00 ± 0.58	3.16 ± 0.26	2.58 ± 0.67	1.98 ± 0.61	1.74 ± 0.48	1.58 ± 0.49	0.97 ± 0.10	0.95 ± 0.50	0.73 ± 0.19	0.78 ± 0.11	0.98 ± 0.18	2.58	1.43	2.86	
18:1 (*n*-9)	11.73 ± 0.22	8.85 ± 0.90	8.89 ± 0.21	9.15 ± 0.83	9.64 ± 0.89	8.31 ± 0.19	10.74 ± 1.81	13.89 ± 1.88	12.29 ± 0.93	10.77 ± 1.87	10.26 ± 1.08	12.38 ± 0.97	12.25 ± 1.12		4.29	25.7	
18:1 (*n*-7)	0.76 ± 0.14	0.60 ± 0.18	0.67 ± 0.17	0.95 ± 0.08	0.79 ± 0.17	1.15 ± 0.30	0.60 ± 0.07	0.61 ± 0.15	0.60 ± 0.10	0.44 ± 0.24	0.47 ± 0.13	0.35 ± 0.08	0.34 ± 0.04				
18:2 (*n*-6)	7.28 ± 0.13	9.05 ± 0.79	8.89 ± 0.68	7.47 ± 0.21	7.97 ± 0.68	5.49 ± 0.23	7.29 ± 0.28	4.88 ± 0.22	4.63 ± 0.27	7.15 ± 0.15	6.59 ± 0.07	7.75 ± 0.28	9.57 ± 0.51	19.4	21.4	4.29	
18:2 (*n*-4)	0.77 ± 0.02	1.43 ± 0.11	1.51 ± 0.14	1.31 ± 0.11	1.35 ± 0.13	1.34 ± 0.39	2.32 ± 0.39	1.19 ± 0.17	1.17 ± 0.12	1.27 ± 0.11	1.06 ± 0.10	1.38 ± 0.06	1.12 ± 0.07				
18:3 (*n*-6)	nd	nd	nd	nd	nd	nd	nd	nd	nd	nd	nd	nd	nd				
18:3 (*n*-4)	nd	nd	nd	nd	nd	nd	nd	nd	nd	nd	nd	nd	nd				
18:3 (*n*-3)	4.26 ± 0.16	4.34 ± 0.60	3.62 ± 0.85	3.40 ± 0.35	3.40 ± 0.61	3.13 ± 0.66	3.07 ± 0.46	2.64 ± 0.06	3.10 ± 0.25	5.35 ± 0.45	4.73 ± 0.21	3.54 ± 0.25	3.76 ± 0.40	52.3	54.3	2.86	
18-4(*n*-3) SDA	7.24 ± 0.59	6.60 ± 0.49	5.70 ± 1.40	4.27 ± 0.18	4.95 ± 1.25	6.03 ± 1.99	5.93 ± 1.36	3.95 ± 0.16	4.86 ± 0.73	10.88 ± 1.58	9.29 ± 1.39	6.09 ± 0.93	6.51 ± 1.09			1.43	
20:0	0.41 ± 0.07	0.55 ± 0.02	0.61 ± 0.08	0.63 ± 0.05	0.75 ± 0.03	0.31 ± 0.01	0.36 ± 0.02	nd	nd	nd	nd	0.35 ± 0.01	0.52 ± 0.03				
20:1 (*n*-11) + (*n*-9)	0.23 ± 0.03	0.65 ± 0.08	0.77 ± 0.21	1.04 ± 0.11	0.93 ± 0.20	0.74 ± 0.33	0.43 ± 0.19	0.40 ± 0.14	0.34 ± 0.16	nd	nd	nd	nd			1.43	
20-1 (*n*-7)	nd	nd	nd	0.09 ± 0.02	0.31 ± 0.05	0.68 ± 0.51	0.14 ± 0.02	0.25 *	0.20 ± 0.03	nd	nd	nd	nd				
20:2 (*n*-6)	0.18 *	0.30 ± 0.06	0.56 ± 0.34	0.86 ± 0.06	0.51 ± 0.32	0.35 ± 0.07	0.30 ± 0.00	0.22 ± 0.02	0.19 ± 0.00	0.08 ± 0.04	0.14 ± 0.02	0.20 ± 0.01	0.17 ± 0.00				
20:3 (*n*-6)	0.50 ± 0.02	0.44 ± 0.02	0.47 ± 0.04	0.36 ± 0.02	0.41 ± 0.03	0.36 ± 0.06	0.47 ± 0.08	0.41 ± 0.04	0.42 ± 0.03	0.37 ± 0.03	0.38 ± 0.02	0.43 ± 0.03	0.59 ± 0.03				
20:4 (*n*-6) ARA	9.85 ± 0.03	13.13 ± 0.65	13.13 ± 1.46	8.88 ± 1.01	10.79 ± 2.66	9.43 ± 1.76	13.38 ± 1.64	9.67 ± 1.12	8.33 ± 1.27	10.64 ± 1.17	10.87 ± 1.59	10.58 ± 1.48	10.81 ± 0.27				
20:3 (*n*-3)	0.91 ± 0.07	0.73 ± 0.20	0.81 ± 0.26	0.69 ± 0.09	0.72 ± 0.14	0.49 *	0.37 *	0.32 ± 0.02	0.28 ± 0.02	0.23 ± 0.05	0.21 ± 0.04	0.26 ± 0.02	0.30 ± 0.07				
20:4 (*n*-3)	nd	0.91 *	0.70 *	0.45 *	0.36 *	0.58 ± 0.11	0.59 ± 0.04	0.27 ± 0.02	0.30 ± 0.06	0.56 ± 0.08	0.50 ± 0.08	0.57 ± 0.13	0.76 ± 0.01				
20:5 (*n*-3) EPA	12.32 ± 0.89	13.48 ± 0.61	11.80 ± 2.11	10.20 ± 0.90	10.22 ± 2.29	11.98 ± 2.40	9.61 ± 1.69	4.70 ± 0.55	6.78 ± 0.55	11.18 ± 1.44	9.69 ± 1.04	9.65 ± 1.53	9.54 ± 1.27			7.14	33.1
22:1 (*n*-11)	0.35 ± 0.10	0.41 ± 0.02	0.58 ± 0.08	0.43 ± 0.06	0.51 ± 0.04	0.18 ± 0.09	0.19 ± 0.04	0.55 ± 0.11	0.34 ± 0.04	0.30 ± 0.12	0.30 ± 0.13	0.50 ± 0.19	0.56 ± 0.33			1.43	
21:5 (*n*-3)	nd	0.68 *	0.15 ± 0.03	0.61 ± 0.06	0.48 ± 0.14	0.18 ± 0.00	0.05 ± 0.01	0.30 ± 0.17	0.41 ± 0.04	0.12 ± 0.03	0.13 ± 0.00	nd	nd				
22:5 (*n*-3)	0.28 ± 0.07	0.25 ± 0.04	0.75 *	0.69 ± 0.08	0.60 ± 0.08	0.55 ± 0.09	0.62 ± 0.13	0.35 ± 0.05	0.34 ± 0.01	0.10 ± 0.05	0.12 ± 0.02	0.17 ± 0.04	0.11 ± 0.03			2.86	
22:6 (*n*-3) DHA	1.54 ± 0.72	4.81 ± 0.15	4.65 ± 2.22	6.95 ± 2.20	4.74 ± 1.99	5.50 ± 0.85	2.74 ± 0.43	1.11 ± 0.24	1.44 ± 0.12	0.81 ± 0.11	0.90 ± 0.14	0.88 ± 0.13	0.61 ± 0.03			18.6	33.1
24:1 (*n*-9)	0.32 ± 0.08	0.37 ± 0.04	0.28*	0.25 ± 0.02	0.40 ± 0.11	nd	nd	0.43 ± 0.08	0.52 ± 0.03	0.13 ± 0.01	0.13 ± 0.01	0.28 ± 0.03	0.28 ± 0.04			1.43	
*∑ IFA*	94.56 ± 0.33 **^abc^**	95.90 ± 1.45	94.58 ± 1.35 **^a^**	92.54 ± 1.26	93.54 ± 2.11 **^ab^**	88.76 ± 1.95	90.08 ± 0.99 **^bc^**	88.76 ± 2.12	87.02 ± 0.64 **^c^**	92.69 ± 0.50	91.98 ± 0.75 **^b^**	90.43 ± 1.73	92.83 ± 2.26 **^a^**				
*n*-6/*n*-3 ratio	0.67 ± 0.03	0.80 ± 0.08	0.85 ± 0.04	0.66 ± 0.08	0.84 ± 0.16	0.56 ± 0.01	0.96 ± 0.07	1.18 ± 0.10	0.78 ± 0.07	0.63 ± 0.04	0.71 ± 0.04	0.91 ± 0.07	1.01 ± 0.12 (**SxM**)				
*EPA + DHA*	13.87 ± 0.17 **^ab^**	16.69 ± 1.93	16.45 ± 0.61 **^a^**	17.15 ± 3.04	14.95 ± 3.32 **^ab^**	17.47 ± 1.55	12.35 ± 1.48 **^a^**	5.82 ± 0.78	8.22 ± 0.58 **^c^**	12.00 ± 1.34	10.59 ± 0.92 **^b^**	10.52 ± 1.55	10.15 ± 1.25 **^b^**				

Data expressed as mean ± standard error (*n* = 3). Different letters in the same row (a, b and c) indicate significant differences (*p* < 0.05) for total identified fatty acids (∑ IFA), *n*-6/*n*-3 ratio and eicosapentaenoic (EPA) + docosahexaenoic acid (DHA) between sampling months; SxM, interaction of the factors site and month; nd, not detected; * Detected in only one of the tree analysed replicates; ^1^ Fatty acid composition (% FAME) of raw cabbage (*Brassica oleracea*), lettuce (*Lactuca sativa*), salmon (*Salmo salar*) and cod (*Gadus morhua*) reported by Foodcomp [29].

**Figure 5:**
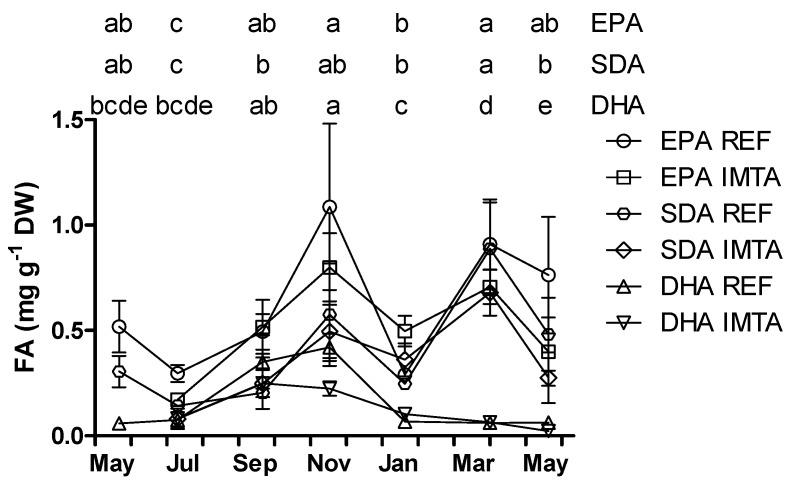
Year-round variation of eicosapentaenoic acid (EPA), docosahexaenoic acid (DHA) and stearidonic acid (SDA) concentration (mg·g^−1^ DW) of *Saccharina latissima* cultivated at a reference site (REF) and at an integrated multi-trophic aquaculture (IMTA) in 2013–2014. Biomass cleaned of epiphytes when present (July–November). The standard errors are presented as bars (*n* = 3), and different letters in the same row indicate significant differences between sampling months (*p* < 0.05).

Estimations of the fatty acid content in mg·g^−1^ showed that EPA content varied significantly from a minimum of 0.17–0.30 mg·g^−1^ DW in July to a maximum of 0.80–1.09 mg·g^−1^ DW in November (Figure 5). The lowest SDA content was found in July (0.08–0.14 mg·g^−1^ DW) and increased significantly up to 0.68–0.89 mg·g^−1^ DW in March (*p* < 0.05). The highest content of DHA was found in September–November (0.22–0.42 mg·g^−1^ DW), while significantly lower content was found in all the other months (0.02–0.10 mg·g^−1^ DW).

Changes in the fatty acid composition were driven by season (*p* < 0.05) while site did not show a significant effect. The fatty acid composition in January was significantly different from all the other sampling months. Moreover, compositions in March and May 2014 were significantly different compared to July, September and November, but similar to May 2013. The similarity percentage (SIMPER) analysis showed that dissimilarities (average squared distance between samples) found in January compared to all the other months were mainly explained by changes in the relative abundance of 20:5*n*-3 (13.12%–33.35%), 14:0 (11.07%–29.37%) and 18:1*n*-9 (10.15%–16.94%). 18:4*n*-3, 14:0 and 22:6*n*-3 explained 20.07%–21.83%, 14.29%–18.75% and 11.76%–20.15%, respectively, of the dissimilarities found in March compared to July, September and November. 14:0, 22:6*n*-3 and 18:1*n*-9 explained 14.11%–21.58%, 13.72%–27.34% and 11.84%–17.25%, respectively, of the dissimilarities observed in May 2014 compared to July, September and November.

Data on the fatty acid composition of *S. latissima* including epiphytes, from July to November, are presented in Table 2. The total identified fatty acids, MUFA and *n*-6 (% FAME) did not vary significantly with season or location (*p* > 0.05). There were significant seasonal changes in the lipid content (% DW), SFA, PUFA, *n*-3, *n*-6/*n*-3 ratio and EPA + DHA but not between cultivation sites. The highest *n*-3 was found in November (27.5%–28.5% FAME) and the highest combined EPA and DHA was found in July and November (8.6%–11.1% FAME).

**Table 2:** Fatty acid composition (% FAME) of *S. latissima* cultivated at both reference (REF) and integrated multi-trophic aquaculture (IMTA) sites, including epiphytes (July–November).

Fatty Acid	July		September		November		FM ^1^	
	REF	IMTA	REF	IMTA	REF	IMTA		
14:0	7.47 ± 1.40	7.63 ± 0.50	10.09 ± 0.83	8.81 ± 1.37	6.74 ± 0.56	5.90 ± 1.29	4.9
14:1	0.29 ± 0.13	0.36 ± 0.11	0.42 ± 0.09	0.76 ± 0.50	0.40 ± 0.17	0.32 ± 0.04	
15:0	0.55 ± 0.03	0.56 ± 0.04	0.48 ± 0.03	0.65 ± 0.20	0.50 ± 0.07	0.44 ± 0.03	
16:0	19.72 ± 1.19	18.30 ± 0.91	19.82 ± 1.48	20.28 ± 1.75	18.28 ± 0.92	15.65 ± 0.64	14.8
16:1 (*n*-7)	5.68 ± 0.24	6.71 ± 0.30	6.90 ± 0.41	6.57 ± 0.04	5.84 ± 0.19	4.73 ± 0.27	5.8
16:2 (*n*-4)	0.44 ± 0.24	0.38 ± 0.14	0.25 ± 0.04	0.55 ± 0.21	0.63 ± 0.07	0.48 ± 0.03	
16:3 (*n*-4)	0.37 ± 0.13	0.50 ± 0.14	0.48 ± 0.05	0.40 ± 0.02	0.55 ± 0.16	0.59 ± 0.22	
17:0	0.72 ± 0.21	0.78 ± 0.03	0.30 ± 0.06	0.64 ± 0.38	0.76 ± 0.08	0.97 ± 0.34	
16:4 (*n*-1)	0.70 ± 0.31	4.84 ± 0.34	0.64 ± 0.28	1.16 ± 0.12	1.75 ± 0.59	2.48 ± 1.12	
18:0	4.28 ± 1.60	3.19 ± 0.27	2.21 ± 0.29	4.16 ± 1.95	2.82 ± 0.23	3.57 ± 0.97	2.1
18:1 (*n*-9)	8.37 ± 1.02	6.52 ± 0.14	10.80 ± 0.72	10.43 ± 0.83	8.39 ± 0.67	7.43 ± 0.80	14.4
18:1 (*n*-7)	1.54 ± 0.57	2.09 ± 0.10	0.92 ± 0.09	1.27 ± 0.51	1.78 ± 0.05	1.52 ± 0.33	
18:2 (*n*-6)	5.15 ± 1.82	4.26 ± 0.25	7.04 ± 0.99	5.87 ± 1.75	3.55 ± 0.27	3.53 ± 0.98	
18:2 (*n*-4)	0.66 ± 0.18	0.65 ± 0.07	1.20 ± 0.34	1.04 ± 0.40	0.76 ± 0.13	0.92 ± 0.41	
18:3 (*n*-6)	nd	nd	nd	nd	nd	nd	
18:3 (*n*-4)	nd	nd	nd	nd	nd	nd	
18:3 (*n*-3)	1.99 ± 0.51	1.82 ± 0.11	1.56 ± 0.29	1.63 ± 0.13	2.34 ± 0.08	2.22 ± 0.71	
18-4 (*n*-3) SDA	2.69 ± 0.75	2.34 ± 0.29	1.88 ± 0.47	1.81 ± 0.42	4.16 ± 0.22	3.97 ± 1.57	
20:0	0.64 ± 0.17	0.37 ± 0.03	0.85 ± 0.05	0.77 ± 0.05	0.29 ± 0.02	0.35 ± 0.06	
20:1 (*n*-11) + (*n*-9)	1.44 ± 0.68	1.42 ± 0.28	0.41 ± 0.17	1.34 ± 0.95	1.12 ± 0.19	0.90 ± 0.25	10.9
20-1 (*n*-7)	0.82 ± 0.18	1.72 ± 0.41	0.15 ± 0.05	0.22 ± 0.11	0.70 ± 0.13	0.53 ± 0.28	
20:2 (*n*-6)	0.32 ± 0.09	0.48 ± 0.19	0.51 ± 0.02	0.41 ± 0.02	0.53 ± 0.01	0.75 ± 0.02	
20:3 (*n*-6)	0.57 ± 0.21	0.66 ± 0.45	0.36 ± 0.07	0.58 ± 0.13	0.36 ± 0.09	0.28 ± 0.08	
20:4 (*n*-6) ARA	6.54 ± 1.61	5.38 ± 0.29	8.60 ± 2.07	7.52 ± 2.60	4.61 ± 0.45	6.76 ± 2.19	
20:3 (*n*-3)	nd	nd	nd	nd	nd	nd	
20:4 (*n*-3)	0.59 ± 0.08	0.28 ± 0.02	0.37 ± 0.02	0.28 ± 0.05	0.58 ± 0.05	0.71 ± 0.26	
20:5 (*n*-3) EPA	9.83 ± 0.61	8.18 ± 0.28	5.60 ± 0.79	5.27 ± 0.65	10.68 ± 1.13	10.87 ± 0.74	10.1
22:1 (*n*-11)	0.28 ± 0.12	0.28 ± 0.07	0.24 ± 0.11	0.56 ± 0.09	0.24 ± 0.08	0.25 ± 0.09	11.9
21:5 (*n*-3)	0.32 ± 0.14	0.61 ± 0.04	0.11 ± 0.04	nd	0.37 ± 0.13	0.24 ± 0.05	
22:5 (*n*-3)	0.88 ± 0.44	0.63 ± 0.07	0.43 ± 0.01	0.53 ± 0.11	0.92 ± 0.15	1.92 ± 0.69	
22:6 (*n*-3) DHA	5.72 ± 1.55	7.42 ± 0.50	3.77 ± 0.06	3.72 ± 0.15	8.27 ± 1.84	8.40 ± 2.18	15.4
24:1 (*n*-9)	nd	nd	nd	nd	nd	nd	
Lipids (% DW)	1.67 ± 0.21	1.70 ± 0.25 ^b^	2.00 ± 0.05	2.68 ± 0.40 ^ab^	2.69 ± 0.17	3.13 ± 0.12 ^a^	
∑ IFA	88.36 ± 1.83	86.30 ± 1.56	86.35 ± 1.58	87.00 ± 1.19	87.74 ± 0.44	86.43 ± 2.31 n.s.	
SFA	33.17 ± 1.20	30.70 ± 1.44 ^a^	33.74 ± 2.72	35.06 ± 2.72 ^a^	29.29 ± 1.54	26.76 ± 0.93 ^b^	
MUFA	18.41 ± 1.82	19.09 ± 1.30	19.83 ± 1.03	21.15 ± 1.32	18.46 ± 1.20	15.68 ± 0.18 n.s.	
PUFA	36.78 ± 3.01	36.50 ± 1.34 ^ab^	32.78 ± 5.32	30.79 ± 5.22 ^b^	40.00 ± 3.15	43.99 ± 2.2 ^a^	
*n*-3	22.14 ± 0.76	20.97 ± 0.56 ^b^	13.71 ± 1.56	13.41 ± 0.89 ^c^	27.46 ± 3.21	28.45 ± 0.7 ^a^	
*n*-6	12.47 ± 3.33	10.78 ± 1.01	16.51 ± 3.16	14.25 ± 4.35	8.86 ± 0.62	11.07 ± 2.99 n.s.	
*n*-6/*n*-3 ratio	0.57 ± 0.17	0.52 ± 0.06 ^b^	1.19 ± 0.09	1.03 ± 0.28 ^a^	0.34 ± 0.07	0.39 ± 0.10 ^b^	
EPA + DHA	10.05 ± 0.49	8.58 ± 0.35 ^a^	5.70 ± 0.75	5.27 ± 0.65 ^b^	11.04 ± 1.25	11.11 ± 0.69 ^a^	

Data expressed as mean ± standard error (*n* = 3); Different letters in the same row (a, b and c) indicate significant differences (*p* < 0.05) for total lipids (% DW), total identified fatty acids (∑ IFA), saturated fatty acid (SFA), monounsaturated fatty acid (MUFA), polyunsaturated fatty acid (PUFA), *n*-3, *n*-6, *n*-6/*n*-3 ratio and eicosapentaenoic (EPA) + docosahexaenoic acid (DHA) between sampling months; n.s., not significant; nd, not detected; ^1^ Fatty acid composition (% FAME) of fish meal from herring (average values for capelin, mackerel and herring) [30].

**Figure 6:**
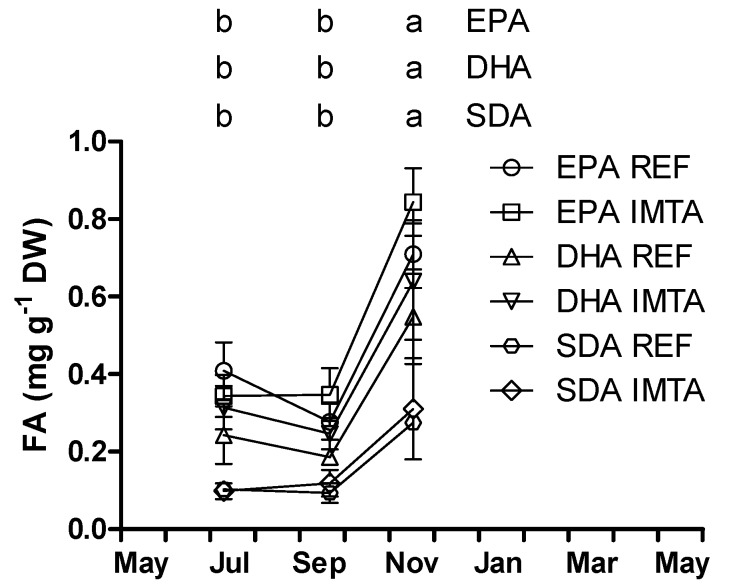
Eicosapentaenoic acid (EPA), docosahexaenoic acid (DHA) and stearidonic acid (SDA) concentration (mg·g^−1^ DW) of *Saccharina latissima* including epiphytes (July–November) cultivated at a reference site (REF) and at an integrated multi-trophic aquaculture (IMTA) in 2013–2014. The standard errors are presented as bars (*n* = 3), and different letters in the same row indicate significant differences between sampling months (*p* < 0.05).

*S. latissima* including epiphytes harvested in November presented significantly higher content of EPA (0.71–0.84 mg·g^−1^ DW), DHA (0.55–0.64 mg·g^−1^ DW) and SDA (0.27–0.31 mg·g^−1^ DW) compared to July and September; 0.28–0.41, 0.19–0.31 and 0.09–0.12 mg·g^−1^ DW, respectively (*p* < 0.05; Figure 6).

The 3-factor analysis of variance including month (July, September and November), site (REF and IMTA) and epiphytic coverage (including epiphytes and cleaned of epiphytes) revealed that the presence of epiphytes and cultivation site did not significantly change the composition of EPA (% FAME); however, seasonal differences occurred (data not shown). There was an interaction between the factors time and epiphytes for DHA, which was significantly higher in the samples including epiphytes but only in November. The MUFA, PUFA and *n*-6 did not change significantly irrespectively of season, cultivation site or presence of epiphytes (*p* > 0.05). The SFA did not change significantly between cultivation sites, but it was significantly higher in July and September compared to November and for the samples including epiphytes compared to the cleaned samples. Changes in the *n*-3 composition were due to an interaction between the factors time and epiphytes, being significantly higher in the cleaned samples compared to the samples including epiphytes but only in July and September. On the other hand, seasonal changes in the *n*-3 composition were only significant for the seaweed samples including epiphytes.

## 3. Discussion

The seasonal lipid content found in this study is in accordance with the values previously reported for *Saccharina latissima* [12] but also for other brown as well as red and green species [12,31,32]. Lipid content of seaweed has been demonstrated to increase in winter and decrease in summer [33], and our results corroborated this pattern.

The relative abundance of SFA, MUFA and PUFA found herein follows the pattern previously reported for *S. latissima* harvested in May and June where PUFA constituted most of the fatty acids (41%–50%), followed by SFA (34%–33%) and MUFA (25%–17%) [10,12] but different from that of the sample harvested in November, where SFA dominated (57%) [10]. As reported for other brown seaweed, *S. latissima* presented high proportions of LC-PUFA’s, especially C18 and C20. Higher proportion of PUFA than SFA found in this study follows the pattern typically observed for brown seaweeds from cold water regions, while in seaweed from warmer waters, the SFA is generally higher [13,34,35]. Increased desaturation of fatty acids has been described as an adaptation to cold water conditions to maintain cell membrane fluidity [36,37], which has been described for other organisms as well [38]. In fact, increased desaturation of fatty acids has been observed during winter/cold season in several brown seaweeds [39]. However, in the present study the lowest PUFA was found in January (winter). Average water temperature in the cultivation sites was 16–16.6 °C, 13.7–13.8 °C, 4.1–4.3 °C and 5.6–5.9 °C in June–August, September–November, December–February and March–May, respectively [18]. Our data are yet in agreement with that reported by Schmid *et al.* [10] who found a marked decrease in the PUFA content of *S. latissima* from June to November. This ability that e.g., algae can change their metabolism to switch on the pathway to utilize the SFA to build up PUFA are unique compared to mammals. The only way for mammals to increase their internal concentration of PUFA is to change their diet with increased PUFA or the very precursors of these [26].

The major SFA and MUFA are 16:0 and 18:1*n*-9 in most studied seaweeds from all the three major groups: green, red and brown [10,12,14]. Of the brown seaweeds, ARA (20:4*n*-6), EPA (20:5*n*-3) and linoleic acid (18:2*n*-6) are generally the most dominant PUFA [10,35,40], while SDA is also found in high proportion [12,32]. In this study, fatty acids containing between 14 and 22 carbons were identified. Concordantly, with that reported for brown seaweeds palmitic acid, oleic acid, ARA and EPA constituted the major fatty acids seasonally/year-round. Furthermore, these results are in accordance with that previously reported for *S. latissima* collected from wild populations in France (March) and Ireland (June and November) [10,12]. In the present study, however, EPA was the major *n*-3 year-round, while in the last mentioned studies, SDA was the most abundant *n*-3 in the samples harvested in March and November. Nevertheless, SDA was also present in significant proportion in the present study. It is noteworthy that DHA, which is generally not found in seaweed or only present in very low proportion [35,40,41], was found in relatively high proportion in this study for samples harvested in July–November (2.7%–6.9%).

The fatty acid composition changed seasonally while location did not affect the fatty acid composition. Based on that composition three distinct groups were identified: winter (January), spring (March and May 2014) and summer/autumn (July, September and November). This data corroborate several previous studies reporting seasonal changes in the fatty acid composition of seaweed [11,41,42]. Similarity in the fatty acid composition between cultivation sites may be explained by the similar environmental conditions found in the two areas including water temperature, salinity, irradiance and inorganic nitrogen and phosphorus. Moreover, the release of inorganic nutrients from the fish farm at the IMTA site was considered negligible compared to the naturally occurring background concentrations of nitrogen [18]. Indeed, the N content was similar or even higher, depending on the harvest time, in the reference site compared to the IMTA site. The utilization of fish farm derived nutrients by seaweed is strongly influenced by a number of factors including nutrient loading, current speed and direction, and distance to the nutrient source. In the present study, seaweed cultivated at the IMTA site was located 500 m from the fish farm, being downstream the fish farm only 50% of the time. Furthermore, the fish farm tonnage was relatively low compared with other commercial salmonid farms [18].

PUFA/SFA ratio is a main indicator to consider when evaluating lipid quality of foods and its recommended minimum value is 0.45 [43]. That is, below the PUFA/SFA ratio found for *S. latissima* in all sampling times in the present study. According to WHO recommendations dietary *n*-6/*n*-3 ratio should not exceed 10. The *n*-6/*n*-3 ratio of brown seaweed harvested at two different seasons varied from 0.6 to 2.8, including *S. latissima* (1) [10]. The low *n*-6/*n*-3 ratio of *S. latissima* found herein year-round (0.56–1.18) suggests that the consumption of this species may contribute to a more balanced dietary *n*-6/*n*-3 ratio.

*Saccharina latissima* presented fatty acid composition rich in LC-PUFA especially ARA and EPA but also SDA and DHA, which are not found in traditional vegetables such as cabbage and lettuce. Additionally, this species contains higher proportions of ARA, SDA and EPA but lower DHA compared to fat fish (salmon), and higher proportions of ARA and SDA but lower EPA and DHA compared to lean fish (cod). Moreover, it presented nutritionally beneficial PUFA/SFA and *n*-6/*n*-3 ratios. However, the quality and quantity of fatty acids are both essential to determine the potential of foods as sources of PUFA. Considering that fatty acids constitute 24.7% of total lipids in *S. latissima* [12] the concentration of EPA/DHA in the present study varied seasonally from 0.3 to 1.5 mg·g^−1^ DW (0.3 to 2.1 mg·g^−1^ DW including the precursor SDA). Based on the recommended daily intake of 250 mg of EPA/DHA (20:5*n*-3/22:6*n*-3) [44], 1106–1627 g of fresh weight (166–244 g DW) of *S. latissima* harvested in November, where highest concentration of EPA/DHA was found, would be needed to fulfil the recommended intake. Schmid *et al.* [10] estimated that the consumption of 1200 g fresh weight (c. 200 g DW) of *Palmaria palmata* would be needed to fulfill the recommended daily intake (based on a recommended daily intake of 1 g [45]). Moreover, for common fish and seafood consumption of 45 and 1275 g of herring and lobster, respectively, are enough to meet the requirements [45]. In the light of these numbers, intake of fresh weight of *S. latissima* to fulfill the needs for EPA/DHA would not be feasible and extraction of *n*-3 from seaweed would be needed.

The essential fatty acid requirements of rainbow trout can be met by 1% (18:3*n*-3) in the diet, as it can be converted in C-20 and C-22 PUFA, while for turbot, the required dietary *n*-3 LC-PUFA is at least 0.8% [46]. Considering the relative low concentration of fatty acids and that the inclusion of seaweed meal in fish feed is generally limited to 10% [47,48,49], *S. latissima* may not be considered an alternative source of fatty acids to fish meal for fish diets. Compared to fish meal, *S. latissima* (including epiphytes) presents lower proportion of EPA (except in November) and DHA, while ARA and SDA are not found in fish meal. However, seaweed present higher levels of PUFA than land vegetables [11], so *S. latissima* may potentially be used as alternative to some vegetable ingredients in fish diets.

Considering harvest time, January must be avoided if the goal is to obtain seaweed with high PUFA content as the proportion of PUFA, *n*-3 (EPA and DHA) *n*-6 (LA) were significantly lower compared to all the other months. Moreover, if the biomass is to be used as an ingredient or food, then harvest in November and March should result in increased concentrations of *n*-3 due to their higher proportion and higher total lipid content during these months. Biomass harvested in November presented epiphytes on the apical, and oldest, part of the frond which makes it unsuitable for food and thus, utilization of this biomass as ingredient for fish feed would be a potential alternative. The presence of epiphytes generally increased the proportions of SFA and DHA, reduced the *n*-3, and showed no difference in the EPA. However, the remaining biomass (basal portion of the frond) in November as well as the biomass harvested in March may be used for food, currently the main commercial application for *S. latissima* [16,19].

## 4. Experimental Section

### 4.1. Sampling Site

*Saccharina latissima* samples were collected from two commercial cultivation areas by Hjarnø Havbrug A/S outside Horsens Fjord, in the inner Danish waters. The IMTA site was located in As Vig (55°47.529′ N, 10°03.027′ E), approximately 100 m from a blue mussel SmartFarm™ (35 tubes incl. nets) and 500 m from rainbow trout (*Oncorhynchus mykiss*) farm cages (175 t per year). The reference (REF) site was established in a 100-ha seaweed cultivation area (55°49.045′ N 10°06.824′ E) located at approximately 2000 m (outside the nutrient impact range) from the fish cages. Fish production season was from April–May to October–December. On 21 May 2013, prior to the experimental period, some ropes were moved from the REF site to the IMTA site for comparison. For further details, see Marinho *et al.* [18].

### 4.2. Seaweed Sampling

*Saccharina latissima* biomass was sampled bimonthly from May 2013 to May 2014 at both cultivation sites, to determine seasonal variations on the lipid content and fatty acid composition. In each sampling, three droppers were selected at random (haphazard sampling; triplicates) and the biomass growing in the first meter (approximately 1–2-m depth) was harvested. At least ten individuals were haphazardly sampled from three droppers (*n* = 3) from both cultivation sites and their fresh weights recorded. All collected samples were frozen until chemical analysis.

### 4.3. Chemical Analyses

Seaweed samples were freeze-dried and ground to fine powder before chemical analyses, using a Siebtechnik Screening disc mill TS 250. Lipids were extracted with a homogeneous mixture of chloroform, methanol, and water (2:2:1.8), following the method of Bligh and Dyer [50]. The method was modified to use a smaller volume of solvents as described by Iverson *et al.* [51], but the original ratio between chloroform, methanol, and water was maintained. *Ca.* 1 g of dried seaweed was used for lipid extraction. The lipid extracts were used for the subsequent determination of fatty acid composition and lipid content. The lipid content was determined by gravimetry after evaporation of chloroform. Duplicate analyses of each sample were performed.

The fatty acid composition of the oil phases was determined after fatty acid methylation and analysis by Gas Chromatography-Flame Ionization Detector (GC-FID). One hundred microliters toluene, 200 µL heptane with 0.01% (*v*/*v*) butylated hydroxytoluene (BHT) and 100 µL internal standard (C23:0) (2% *w*/*v*) were added to an amount of lipid extract corresponding to 30–60 mg lipid. One mililiters of borontriflouride (BF3) in methanol was added to the lipid extract mixture and the lipids were methylated in a one-step procedure using a microwave oven (Multiwave 3000 SOLV, Anton Paar, Graz, Austria) with a 64MG5 rotor. The settings for the microwave were 5 min at 500 Watt followed by 10 min cooling. The fatty acid methyl esters (FAMEs) were washed with 1 mL saturated NaCl and 0.7 mL heptane with 0.01% (*v*/*v*) BHT. The heptane phase was transferred to a GC vial and FAMEs were analysed by GC (HP 5890A, Agilent Technologies, Palo Alto, CA, USA) according to the American Oil Chemists’ Society (AOCS) [52]. For separation, DB127-7012 column (10 m × ID 0.1 mm × 0.1 µm film thickness, Agilent Technologies, Palo Alto, CA, USA) was used. Injection volume was 0.2 μL in split mode (1:50). The initial temperature of the GC-oven was 160 °C. The temperature was set to increase gradually being as follows: 160–200 °C (10.6 °C min^−1^), 200 °C kept for 0.3 min, 200–220 °C (10.6 °C min^−1^), 220 °C kept for 1 min, 220–240 °C (10.6 °C min^−1^) and kept at 240 °C for 3.8 min. The determination was made in duplicates. Fatty acids were identified by comparison of retention times with those from a mixture of known fatty acid standards. Results were given in area %. Moreover, content of fatty acids in mg·g^−1^ were estimated using data from Fleurence *et al.* [12] suggesting that sum of fatty acids in *S. latissima* constitute 24.7% of total lipids.

### 4.4. Statistical Analysis

Data is expressed as mean ± standard error. A permutational Analysis of Variance (ANOVA) (using Euclidian distances) was used to test the effect of time (random) and site (REF and IMTA; fixed) on all tested response parameters (PERMANOVA package in PRIMER+ [53]; type III sum of squares and unrestricted permutation (9999) on raw data (α = 0.05)). This was followed by a SIMPER analysis (based also on Euclidian distances) to identify those fatty acid species that contributed most to the observed differences among time and/or site.

## 5. Conclusions

Cultivation of *S. latissima* in the present IMTA system did not result in changes in total lipids or fatty acid composition compared to the reference site. However, significant seasonal changes were found in both sites. Harvest time in November and March should resulte in maximum *n*-3 content. The presence of epiphytes at some harvest times generally increased the proportions of SFA and DHA, and reduced the *n*-3. Although *S. latissima* presented relatively low lipid content, and consequently low concentrations of fatty acids, its fatty acid profile contained high proportions of LC-PUFA, especially ARA and EPA but also SDA and DHA. Moreover, *S. latissima* presented nutritionally beneficial PUFA/SFA and *n*-6/*n*-3 ratios. PUFA’s accounted for up to 52.3–54.0 (% FAME) in July. Thus, extraction of *n*-3 and *n*-6 from seaweed should be considered. Compared to traditional vegetables, such as cabbage and lettuce, *S. latissima* presents a better source of LC-PUFA’s. Additionally, compared to fat (salmon) and lean fish (cod) this seaweed species contains higher proportions of ARA and SDA, but lower EPA (only cod) and DHA.

## References

[1] Van Ginneken V.J.T., Helsper J.P.F.G., de Visser W., van Keulen H., Brandenburg W.A. (2011). Polyunsaturated fatty acids in various macroalgal species from North Atlantic and tropical seas. Lipids Health Dis..

[2] Gerster H. (1998). Can adults adequately convert alpha-linolenic acid (18:3*n*-3) to eicosapentaenoic acid (20:5*n*-3) and docosahexaenoic acid (22:6*n*-3)?. Int. J. Vitam. Nutr. Res..

[3] Owen J.M., Adron J.W., Middleton C., Cowey C.B. (1975). Elongation and desaturation of dietary fatty acids in turbot *Scophthalmus maximus* L., and rainbow trout, *Salmo gairdnerii* rich. Lipids.

[4] March B.E. (1993). Essential fatty acids in fish physiology. Can. J. Physiol. Pharmacol..

[5] Lands W., Barlow S., Stansby M. (1982). Nutritional Evaluation of Long Chain Fatty Acids in Fish Oil.

[6] Simopoulos A.P. (2002). The importance of the ratio of omega-6/omega-3 essential fatty acids. Biomed. Pharmacother..

[7] Simopoulos A.P. (2008). The importance of the omega-6/omega-3 fatty acid ratio in cardiovascular disease and other chronic diseases. Exp. Biol. Med..

[8] Bell M.V., Henderson R.J., Sargent J.R. (1986). The role of polyunsaturated fatty acids in fish. Comp. Biochem. Physiol. B..

[9] Wahbeh M.I. (1997). Amino acid and fatty acid profiles of four species of macroalgae from Aqaba and their suitability for use in fish diets. Aquaculture.

[10] Schmid M., Guihéneuf F., Stengel D.B. (2014). Fatty acid contents and profiles of 16 macroalgae collected from the Irish Coast at two seasons. J. Appl. Phycol..

[11] Kendel M., Couzinet-Mossion A., Viau M., Fleurence J., Barnathan G., Wielgosz-Collin G. (2013). Seasonal composition of lipids, fatty acids, and sterols in the edible red alga *Grateloupia turuturu*. J. Appl. Phycol..

[12] Fleurence J., Gutbier G., Mabeau S., Leray C. (1994). Fatty acids from 11 marine macroalgae of the French Brittany coast. J. Appl. Phycol..

[13] Colombo M.L., Risè P., Giavarini F., De Angelis L., Galli C., Bolis C.L. (2006). Marine macroalgae as sources of polyunsaturated fatty acids. Plant Foods Hum. Nutr..

[14] Kumari P., Kumar M., Gupta V., Reddy C.R.K., Jha B. (2010). Tropical marine macroalgae as potential sources of nutritionally important PUFAs. Food Chem..

[15] Food and Agriculture Organization (FAO) of the United Nations Fishery Statistical Collections. Global Aquaculture Production. http://www.fao.org/fishery/statistics/global-aquaculture-production/en.

[16] Peteiro C., Salinas J.M., Freire Ó., Fuertes C. (2006). Cultivation of the autoctonous seaweed *Laminaria saccharina* off the Galician coast (NW Spain): Production and features of the sporophytes for an annual and biennial harvest. Thalassas.

[17] Reid G.K., Chopin T., Robinson S.M.C., Azevedo P., Quinton M., Belyea E. (2013). Weight ratios of the kelps, *Alaria esculenta* and *Saccharina latissima*, required to sequester dissolved inorganic nutrients and supply oxygen for Atlantic salmon, *Salmo salar*, in Integrated Multi-Trophic Aquaculture systems. Aquaculture.

[18] Marinho G.S., Holdt S.L., Birkeland M.J., Angelidaki I. (2015). Commercial cultivation and bioremediation potential of sugar kelp, *Saccharina latissima*, in Danish waters. J. Appl. Phycol..

[19] Sanderson J.C., Dring M.J., Davidson K., Kelly M.S. (2012). Culture, yield and bioremediation potential of *Palmaria palmata* (Linnaeus) Weber & Mohr and *Saccharina latissima* (Linnaeus) C.E. Lane, C. Mayes, Druehl & G.W. Saunders adjacent to fish farm cages in northwest Scotland. Aquaculture.

[20] Peteiro C., Freire Ó. (2013). Biomass yield and morphological features of the seaweed *Saccharina latissima* cultivated at two different sites in a coastal bay in the Atlantic coast of Spain. J. Appl. Phycol..

[21] Neori A., Chopin T., Troell M., Buschmann A.H., Kraemer G.P., Halling C., Shpigel M., Yarish C. (2004). Integrated aquaculture: Rationale, evolution and state of the art emphasizing seaweed biofiltration in modern mariculture. Aquaculture.

[22] Chopin T., Cooper J.A., Reid G., Cross S., Moore C. (2012). Open-water integrated multi-trophic aquaculture: Environmental biomitigation and economic diversification of fed aquaculture by extractive aquaculture. Rev. Aquac..

[23] Chopin T., Yarish C., Wilkes R., Belyea E., Lu S. (2000). Developing Porphyra/salmon integrated aquaculture for bioremediation and diversification of the aquaculture industry. J. Appl. Phycol..

[24] Chopin T., Robinson S.M.C., Troell M., Neori A., Buschmann A.H., Fang J., Jørgensen S.E., Fath B.D. (2008). Multitrophic integration for sustainable marine aquaculture. Ecological Engineering.

[25] Abreu M.H., Varela D.A., Henríquez L., Villarroel A., Yarish C., Sousa-Pinto I., Buschmann A.H. (2009). Traditional *vs.* integrated multi-trophic aquaculture of *Gracilaria chilensis* C.J. Bird, J. McLachlan & E.C. Oliveira: Productivity and physiological performance. Aquaculture.

[26] Guschina I.A., Harwood J.L. (2006). Lipids and lipid metabolism in eukaryotic algae. Prog. Lipid Res..

[27] Floreto E.A.T., Teshima S., Ishikawa M. (1996). Effects of nitrogen and phosphorus on the growth and fatty acid composition of *Ulva pertusa* Kjellman (Chlorophyta). Bot. Mar..

[28] Floreto E.A.T., Hirata H., Ando S., Yamasaki S. (1993). Effects of temperature, light intensity, salinity and source of nitrogen on the growth, total lipid and fatty acid composition of *Ulva pertusa* Kjellman (Chlorophyta). Bot. Mar..

[29] DTU Food Food Composition Databank. National Food Institute. http://www.foodcomp.dk/v7/fcdb_search.asp.

[30] Opstvedt J. (1985). Fish Lipids in Animal Nutrition.

[31] Wong K.H., Cheung P.C.K. (2000). Nutritional evaluation of some subtropical red and green seaweeds: Part I—Proximate composition, amino acid profiles and some physico-chemical properties. Food Chem..

[32] Dawczynski C., Schubert R., Jahreis G. (2007). Amino acids, fatty acids, and dietary fibre in edible seaweed products. Food Chem..

[33] Nelson M.M., Phleger C.F., Nichols P.D. (2002). Seasonal lipid composition in macroalgae of the northeastern Pacific Ocean. Bot. Mar..

[34] Sánchez-Machado D.I., López-Cervantes J., López-Hernández J., Paseiro-Losada P. (2004). Fatty acids, total lipid, protein and ash contents of processed edible seaweeds. Food Chem..

[35] Silva G., Pereira R.B., Valentão P., Andrade P.B., Sousa C. (2013). Distinct fatty acid profile of ten brown macroalgae. Rev. Bras. Farmacogn..

[36] Moon B.Y., Higashi S., Gombos Z., Murata N. (1995). Unsaturation of the membrane lipids of chloroplasts stabilizes the photosynthetic machinery against low-temperature photoinhibition in transgenic tobacco plants. Proc. Natl. Acad. Sci. USA.

[37] Gombos Z., Wada H., Murata N. (1994). The recovery of photosynthesis from low-temperature photoinhibition is accelerated by the unsaturation of membrane lipids: A mechanism of chilling tolerance. Proc. Natl. Acad. Sci. USA.

[38] White F., Somero G. (1982). Acid-base regulation and phospholipid adaptations to temperature: Time courses and physiological significance of modifying the milieu for protein function. Physiol. Rev..

[39] Pohl P., Zurheide F., Hoppe H.A., Levring T., Tanaka Y. (1979). Fatty acids and lipids of marine algae and the control of their biosynthesis by environmental factors. Marine Algae in Pharmaceutical Science.

[40] Tabarsa M., Rezaei M., Ramezanpour Z., Waaland J.R., Rabiei R. (2012). Fatty acids, amino acids, mineral contents, and proximate composition of some brown seaweeds. J. Phycol..

[41] Polat S., Ozogul Y. (2013). Seasonal proximate and fatty acid variations of some seaweeds from the northeastern Mediterranean coast. Oceanologia.

[42] Iveša L., Blažina M., Najdek M. (2004). Seasonal variations in fatty acid composition of *Caulerpa taxifolia* (M. Vahl.) C. Ag. in the northern Adriatic Sea (Malinska, Croatia). Bot. Mar..

[43] Department of Health (1994). Nutritional Aspects of Cardiovascular Disease. Report Health Social Subjects No. 46.

[44] Lagiou P., Løvik M., Marchelli R., Martin A., Moseley B., Berg H.V.L., Verhagen H. (2009). Labelling reference intake values for *n*-3 and *n*-6 polyunsaturated fatty acids Scientific Opinion of the Panel on Dietetic Products, Nutrition and Allergies on a request from the Commission related to labelling reference intake values for *n*-3 and *n*-6 poly. EFSA J..

[45] Ward O.P., Singh A. (2005). Omega-3/6 fatty acids: Alternative sources of production. Process Biochem..

[46] FAO Fisheries and aquaculture department Lipids and Fatty Acids Aquaculture Development and Coordination Programme. Fish Feed Technology. Proceedings of the FAO/UNDP Training Course in Fish Feed Technology.

[47] Marinho G., Nunes C., Sousa-Pinto I., Pereira R., Rema P., Valente L.M.P. (2013). The IMTA-cultivated Chlorophyta *Ulva* spp. as a sustainable ingredient in Nile tilapia (*Oreochromis niloticus*) diets. J. Appl. Phycol..

[48] Valente L.M.P., Gouveia A., Rema P., Matos J., Gomes E.F., Pinto I.S. (2006). Evaluation of three seaweeds *Gracilaria bursa-pastoris*, *Ulva rigida* and *Gracilaria cornea* as dietary ingredients in European sea bass (*Dicentrarchus labrax*) juveniles. Aquaculture.

[49] Soler-Vila A., Coughlan S., Guiry M.D., Kraan S. (2009). The red alga *Porphyra dioica* as a fish-feed ingredient for rainbow trout (*Oncorhynchus mykiss*): Effects on growth, feed efficiency, and carcass composition. J. Appl. Phycol..

[50] Bligh E.G., Dyer W.J. (1959). A rapid method of total lipid extraction and purification. Can. J. Biochem. Physiol..

[51] Iverson S.J., Lang S.L., Cooper M.H. (2001). Comparison of the Bligh and Dyer and Folch methods for total lipid determination in a broad range of marine tissue. Lipids.

[52] Firestone D., Society A.O.C. (1998). Official Methods and Recommended Practices of the AOCS.

[53] Anderson M., Gorley R., Clarke K. (2008). PERMANOVA+ for PRIMER: Guide to Software and Statistical Methods.

